# Welcome to the new tRNA world!

**DOI:** 10.3389/fgene.2014.00336

**Published:** 2014-09-23

**Authors:** Akio Kanai

**Affiliations:** Functional RNA Group, Institute for Advanced Biosciences, Keio UniversityTsuruoka, Japan

**Keywords:** transfer RNA, molecular biology, gene diversity, pre-tRNA processing, base modification, new biological functions, molecular evolution, human diseases

Transfer RNAs (tRNAs) are one of the classical non-coding RNAs, with lengths of approximately 70–100 bases. The secondary structure of tRNAs can be represented as a cloverleaf with four stems, and the three-dimensional structure as an “L” shape. Historically, the basic function of the tRNAs as essential components of translation was established in the 1960s, when it was found that each tRNA is charged with a target amino acid by a specific aminoacyl-tRNA synthetase, and delivers it to the ribosome during protein synthesis (Crick, 1966; Normanly and Abelson, 1989; Frank, 2000). However, recent studies suggest that the roles of tRNA in cellular regulation go beyond this paradigm. Now, tRNA is recognized as a regulator of many biological processes, and several unique tRNA genes have been discovered. Our understanding of the enzymes involved in tRNA functions has also increased and many tRNA-related diseases have been reported. In response to these exciting data, I have edited this special issue of *tRNA*, which revisits and summarizes the molecular biology of tRNA. The topics contributed by specialists in the field cover a wide range of tRNA research.

In the last decade, a number of reports have described novel aspects of tRNAs in terms of the diversity of their genes. For example, several types of disrupted tRNA genes have been reported in the Archaea and primitive Eukarya. These include multiple-intron-containing tRNA genes, split tRNA genes, and permuted tRNA genes (Fujishima and Kanai, 2014; Soma, 2014). Because these tRNAs are encoded as precursor forms (pre-tRNAs) in the genome, they must be processed to yield mature functional tRNAs. Studies of tRNA introns and their processing enzymes suggest that rather complex pathways are required to generate mature tRNAs (Yoshihisa, 2014). Metazoan mitochondrial tRNA is another example of a unique tRNA, lacking either one or two arms of the typical tRNA cloverleaf structure (Watanabe et al., 2014), and transfer messenger RNA (tmRNA) is involved in *trans*-translation, the major ribosome rescue system in bacterial cells (Himeno et al., 2014). Most of these tRNA genes and a huge number of tRNA sequences from metagenomic analyses are registered on the tRNA gene databases (Abe et al., 2014).

The universal 3′-terminal CCA sequence of the tRNAs, which is required for amino acid attachment to the molecule, is synthesized by tRNA nucleotidyltransferase or the “CCA-adding enzyme.” The molecular mechanism of the template-independent RNA polymerization catalyzed by the CCA-adding enzyme is discussed, based on its structural features (Tomita and Yamashita, 2014). It is well-known that tRNAs contain many types of base modifications. Recent progress in our understanding of two major modifications in tRNAs, methylated nucleosides (Hori, 2014) and thionucleosides (Shigi, 2014), is reviewed and summarized.

As well as the canonical role of tRNA during protein biosynthesis, recent studies have shown that tRNA performs additional functions in regulating biochemical processes (Raina and Ibba, 2014). For example, aminoacyl-tRNA is involved in cell wall formation, protein labeling for degradation, and antibiotic biosynthesis. Moreover, tRNA cleavage is a conserved part of the responses of eukaryotic cells to various stresses. Age-associated and tissue-specific tRNA fragmentation have also been observed and several studies have suggested that some of these tRNA fragments are involved in the cellular RNA interference (RNAi) system.

Pathological mutations in tRNA genes and tRNA-related enzymes have been linked to human diseases (Abbott et al., 2014). Mutations in the mitochondrial tRNA genes, in particular, are responsible for many diseases, and aminoacyl-tRNA synthetase mutations are associated with neurological diseases. Finally, the evolution of the tRNA molecule is discussed based on the analysis of the tRNA structure (Caetano-Anolles and Sun, 2014) and ancestral ribozymes (Fujishima and Kanai, 2014).

Please enjoy reading all these articles, which will open the door to a new tRNA world!

## Conflict of interest statement

The author declares that the research was conducted in the absence of any commercial or financial relationships that could be construed as a potential conflict of interest.
